# Prognostic risk assessment model for alternative splicing events and splicing factors in malignant pleural mesothelioma

**DOI:** 10.1002/cam4.5174

**Published:** 2022-08-28

**Authors:** Yue Jiang, Chengda Zhang, Yang Chen, Shiyu Zhao, Yipeng He, Jun He

**Affiliations:** ^1^ Department of Clinical Medicine Southwest Medical University Luzhou China; ^2^ Department of Gastroenterology The Third Hospital of Mian Yang (Sichuan Mental Health Center) Mianyang China; ^3^ Department of Oncology The Third Hospital of Mian Yang (Sichuan Mental Health Center) Mianyang China

## Abstract

**Background:**

Malignant pleural mesothelioma (MPM) is a rare and highly malignant thoracic tumor. Although alternative splicing (AS) is associated with tumor prognosis, the prognostic significance of AS in MPM is unknown.

**Methods:**

Transcriptomic data, clinical information, and splicing percentage values for MPM were obtained from The Cancer Genome Atlas (TCGA) and TCGA SpliceSeq databases. Least absolute shrinkage and selection operator (LASSO) regression and multivariate Cox analyses were performed to establish a model affecting the prognosis of MPM. Survival and ROC analyses were used to test the effects of the prognostic model. LASSO/multivariate Cox analysis was used to construct the MPM prognostic splicing factor (SF) model. The SF–AS interaction network was analyzed using Spearman correlation and visualized using Cytoscape. The association between the MPM prognostic SF model and drug sensitivity to chemotherapeutic agents such as cisplatin was analyzed using pRRophetic.R.

**Results:**

The LASSO/multivariate Cox analysis identified 41 AS events and 2 SFs that were mostly associated with survival. Nine prognostic prediction models (i.e., seven types of AS model, total AS model, and SF model) were developed. An MPM prognostic SF–AS regulatory network was subsequently constructed with decreased drug sensitivity in the SF model high‐risk group (*p* = 0.025).

**Conclusion:**

This study provides the first comprehensive analysis of the prognostic value of AS events and SFs in MPM. The SF–AS regulatory network established in this study and our drug sensitivity analysis using the SF model may provide novel targets for pharmacological studies of MPM.

## Graphical abstract

Prognostic signatures for alternative splicing events and splicing factors in malignant pleural mesothelioma.

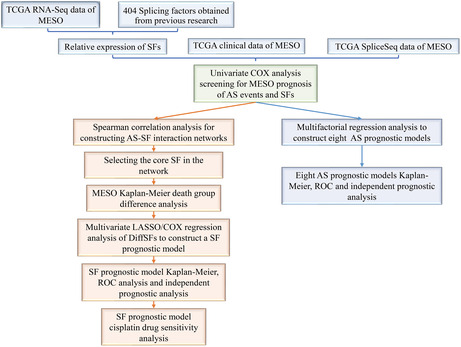

## 1 INTRODUCTION

Malignant pleural mesothelioma (MPM) is a rare and extremely malignant tumor^1^ that is mainly caused by asbestos. Despite the reduced use of asbestos, the mortality rate of patients with MPM is increasing every year.^2^, ^3^ Currently, there are limited treatment alternatives for advanced MPM, and the use of cisplatin/pemetrexed combination improves patient survival by 1–2 months; therefore, there is an urgent need for the development of new pharmacological targets.^3^, ^4^ Alternative splicing (AS) is a common regulatory process in gene expression that produces various mRNA isoforms using different splicing methods.^5^ The occurrence of AS may affect tumor oncogene expression, leading to the development of cancer.^6^, ^7^, ^8^ For example, AS of TrkA regulates the development of human neuroblastoma,^9^ MTR4 drives liver tumorigenesis by promoting cancer metabolic conversion through AS,^10^ and AKAP8 inhibits tumor metastasis by antagonizing EMT‐related AS.^11^ Thus, targeted AS has been newly developed for treating tumors.^12^, ^13^ The proteins that regulate AS are known as splicing factors (SFs). Based on their functional roles, they can be categorized as small nuclear ribonucleoprotein particle (snRNP) and non‐snRNP protein factors.^14^ Furthermore, a single SF may regulate multiple splicing events and play an essential role in tumor development, which is associated with several tumor prognoses.^15^, ^16^, ^17^ To improve MPM prognosis and suggest novel ideas for MPM‐targeted pharmacological experiments, we analyzed MPM data in The Cancer Genome Atlas (TCGA).^18^ TCGA SpliceSeq 2.0^19^ was used via a bioinformatics approach to determine the AS events and SFs with considerable prognostic relevance during MPM development.

## 2 MATERIALS AND METHODS

### 2.1 Data download and preprocessing

RNA‐seq data (FPKM) and clinical information regarding MPM were downloaded from the TCGA database (https://portal.gdc.cancer.gov/)^18^ and TCGA SpliceSeq database 2.0 (https://bioinformatics.mdanderson.org/public‐datasets/).^19^ Samples with PSI values of >75% were selected to download the percentage of AS (PSI) values for MPM. Patients without follow‐up information and those with a survival of <30 days were excluded. The impute.knn function was used to add missing data and remove the samples with PSI values of <0.05 and AS events with standard deviations of <0.01, thus preventing interference from unchanged AS samples. AS events involved the alternative acceptor (AA) sites, alternative donor (AD) sites, alternative promoters (AP), alternative terminators (AT), exon skipping (ES), mutually exclusive exons (ME), and retained introns (RI).

### 2.2 Univariate Cox analysis to screen for prognosis‐related AS events

Univariate Cox analysis was performed for the preliminary screening of prognostic tumor correlates. Clinical data and PSI values obtained from the TCGA database were combined to initially screen AS events and determine overall survival (OS) using a one‐way Cox analysis. The AS events associated with OS (*p* < 0.05) were selected, and the results were visualized using the upset.R package.^20^ We used the ggplot2.R package to clearly demonstrate the association between each AS event and MPM prognosis.^21^ In this package, prognosis‐related AS events were presented as bubble plots and volcano plots. Univariate Cox analysis was used to screen for SFs that might be associated with MPM prognosis.

### 2.3 LASSO regression and multivariate Cox analyses to construct a prognostic model

To investigate each type of AS and the association between SFs and MPM prognosis, we individually modeled the prognosis for each type of AS event (including a total AS model). Overfitting AS events and SFs were excluded using LASSO regression. Multivariate Cox analysis was used to determine the final survival‐related AS events, estimate the OS risk values for MPM, and visualize these events using R. Moreover, it was used to screen for SFs that were strongly associated with MPM prognosis. Further, the MPM samples were stratified according to the AS type and categorized into high‐ and low‐risk groups based on the median risk values. Kaplan–Meier survival analysis^22^ and ROC analysis were performed for both groups to test the validity of the model and reliability of model predictions, respectively.

### 2.4 Analysis of independent prognostic factors

To estimate the prognostic value of the AS model, we analyzed several clinical prognostic factors, including risk scores for sex, pathological stage, T category (to assess the aggressiveness of the tumor), N category (to assess the lymph node metastasis of the tumor), M category (to assess the distant metastasis of the tumor), and markers. These factors were considered as independent prognostic variables when the *p* value was <0.05 in univariate and multivariate analyses.

### 2.5 SF–AS interaction network analysis

We downloaded the currently known 404 SFs^23^ to determine the SF expression values (FPKM) using RNA‐seq expression profiles of patients with MPM. Univariate Cox analysis was performed to screen for prognostic associations with SFs, and Spearman test analysis was used to identify the AS events and SFs that were strongly associated with OS. The SF–AS interaction network was mapped using the Cytoscape software.^24^ When the SF is more connected and centrally located in the network, it is more likely to play a central role in the development of MPM.

### 2.6 Gene set enrichment analysis (GSEA) of SF

To investigate the molecular mechanism of SF affecting MPM prognosis and suggest ideas for subsequent basic experiments, we selected the core SFs in the SF–AS network, used the SF expression values in TCGA data (log2FPKM), categorized the samples into survival and death groups, and used an unpaired *t*‐test to screen for differences in the survival and death of SFs. After LASSO/multivariate Cox analysis, the SF prognostic model of MPM was constructed and tested using survival, ROC, and independent prognostic analyses. The association between the prognostic model and drug sensitivity to cisplatin was tested using the pRRophetic.R package.^25^ Samples were classified into two groups based on their median core SF expression level. The pathways in which the core SF decreased the sensitivity of sensitivity of mesothelioma to drugs were analyzed using the GSEA software.^26^ Major pathways were selected for visualization.

## 3 RESULTS

### 3.1 Primary screening of MPM prognosis‐associated AS events

**FIGURE 1:**
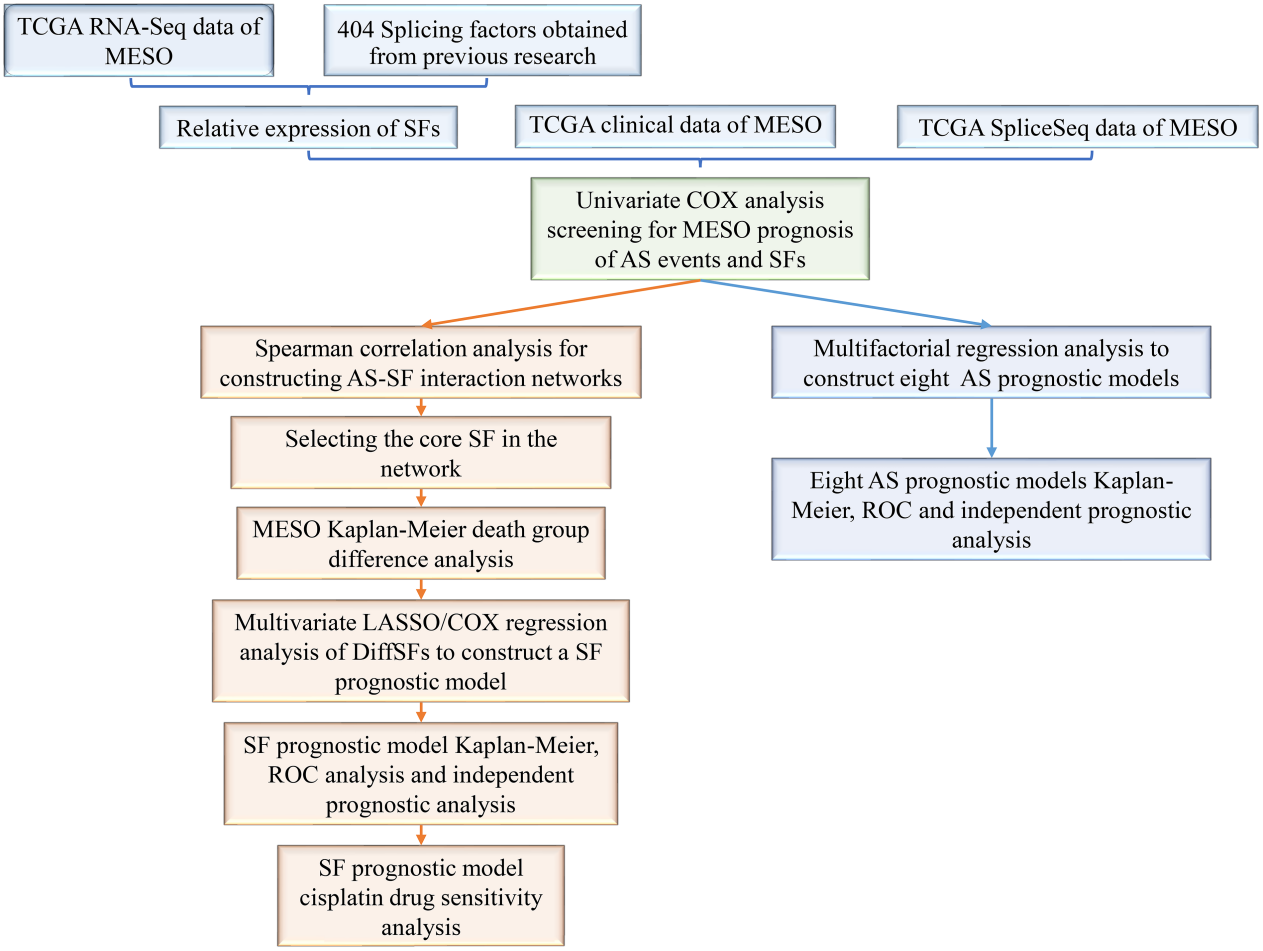
Flowchart. The general flow of the article is shown in the figure. After obtaining MPM data from TCGA and TCGA SpliceSeq databases, AS event modeling was performed to suggest new ideas for MPM research.

**FIGURE 2:**
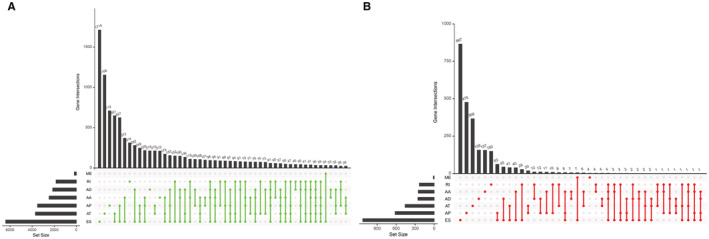
Upset plot for AS events and genes for MPM. Upset plots for all seven types of AS events for MPM (A) and MPM prognostic associations (B).

**FIGURE 3:**
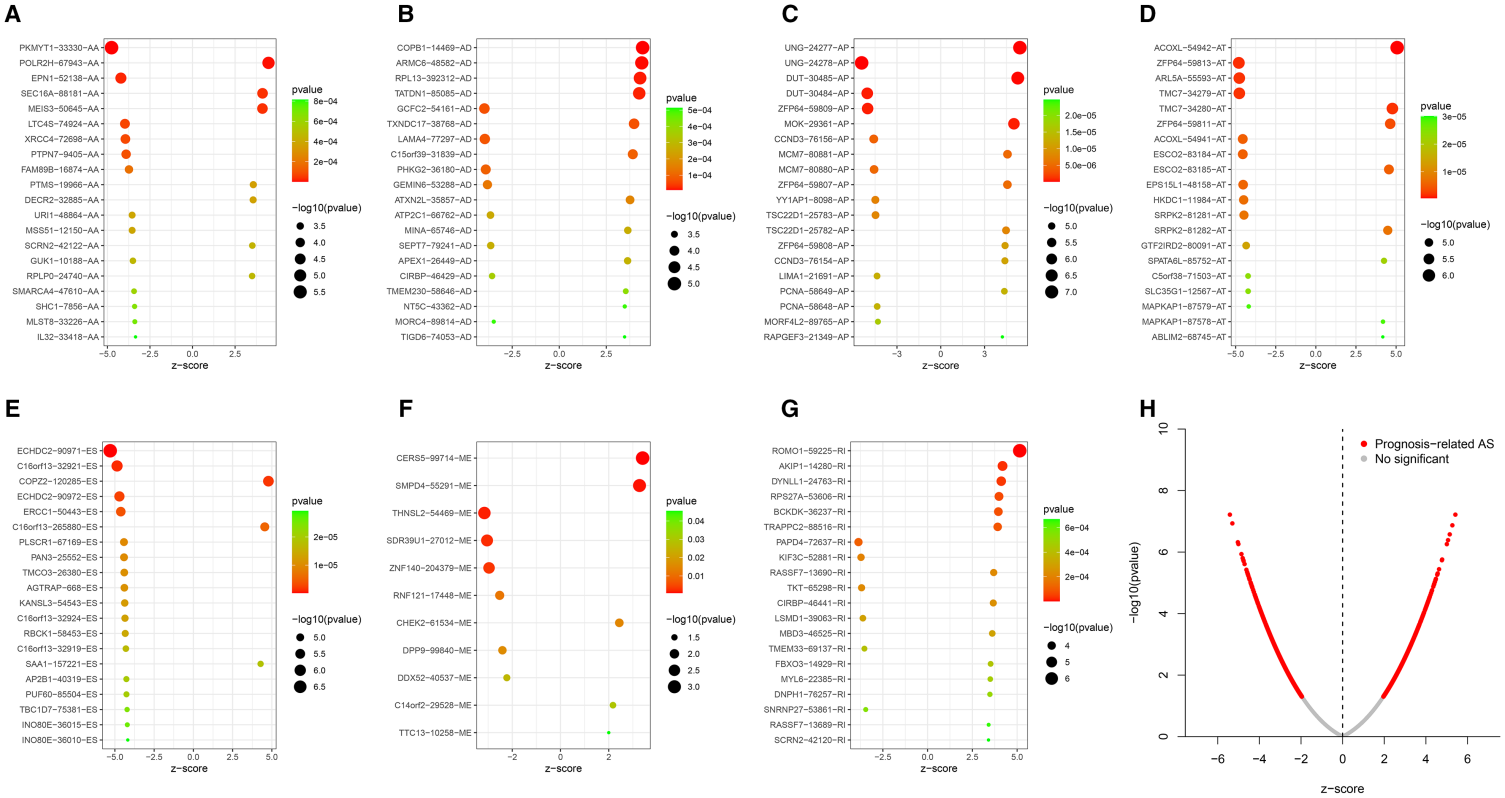
Bubble and volcano plots for MPM prognosis‐associated AS events. The top 10–20 AS events ranked by the *p*‐values of the one‐way Cox analysis for types AA (A), AD (B), AP (C), AT (D), ES €, ME (F), and RI (G). Figure 3H shows a volcano map for prognosis‐related alternate splicing events. AA, alternate acceptor site; AD, alternate donor site; AP, alternate promoter; AT, alternate terminator; ES, exon skipping; ME, mutually exclusive exon; RI, retained intron.

RNA‐seq expression profiles and clinical information of 87 samples were obtained from the TCGA official website, and data on 43,433 cross‐splicing events were obtained from the TCGA SpliceSeq 2.0 database. A general flowchart is shown in Figure 1. The association of genes with cross‐splicing types is represented using an upset plot (Figure 2A). In total, 84 samples and 28,664 AS events remained after filtering samples with a total survival time of <30 days using R. A total of 3974 prognosis‐associated crossover splicing events were initially screened using the univariate Cox analysis. The upset plot showed that multiple crossover splicing events could occur for each gene. The most common type of crossover splicing was ES, whereas the least common type was ME (Figure 2B). The top 10–20 AS events were selected using the *p*‐values of one‐way Cox analysis and were presented using a volcano plot to clearly demonstrate the prognostic risk of various AS events in patients with MPM (Figure 3).

### 3.2 Eight MPM prognosis AS models

**FIGURE 4:**
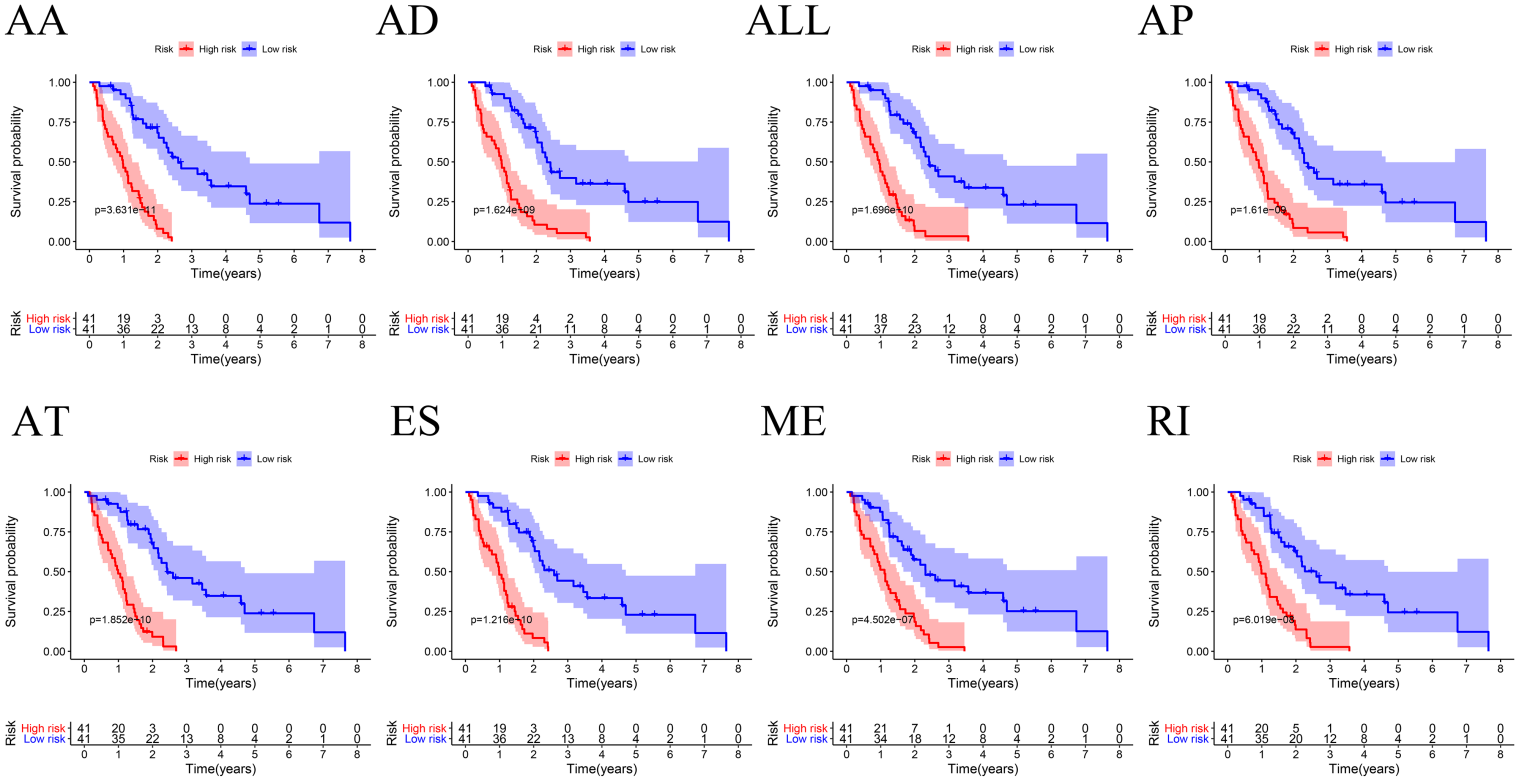
Kaplan–Meier curves for OS between patients in the high‐ and low‐risk groups.

**FIGURE 5:**
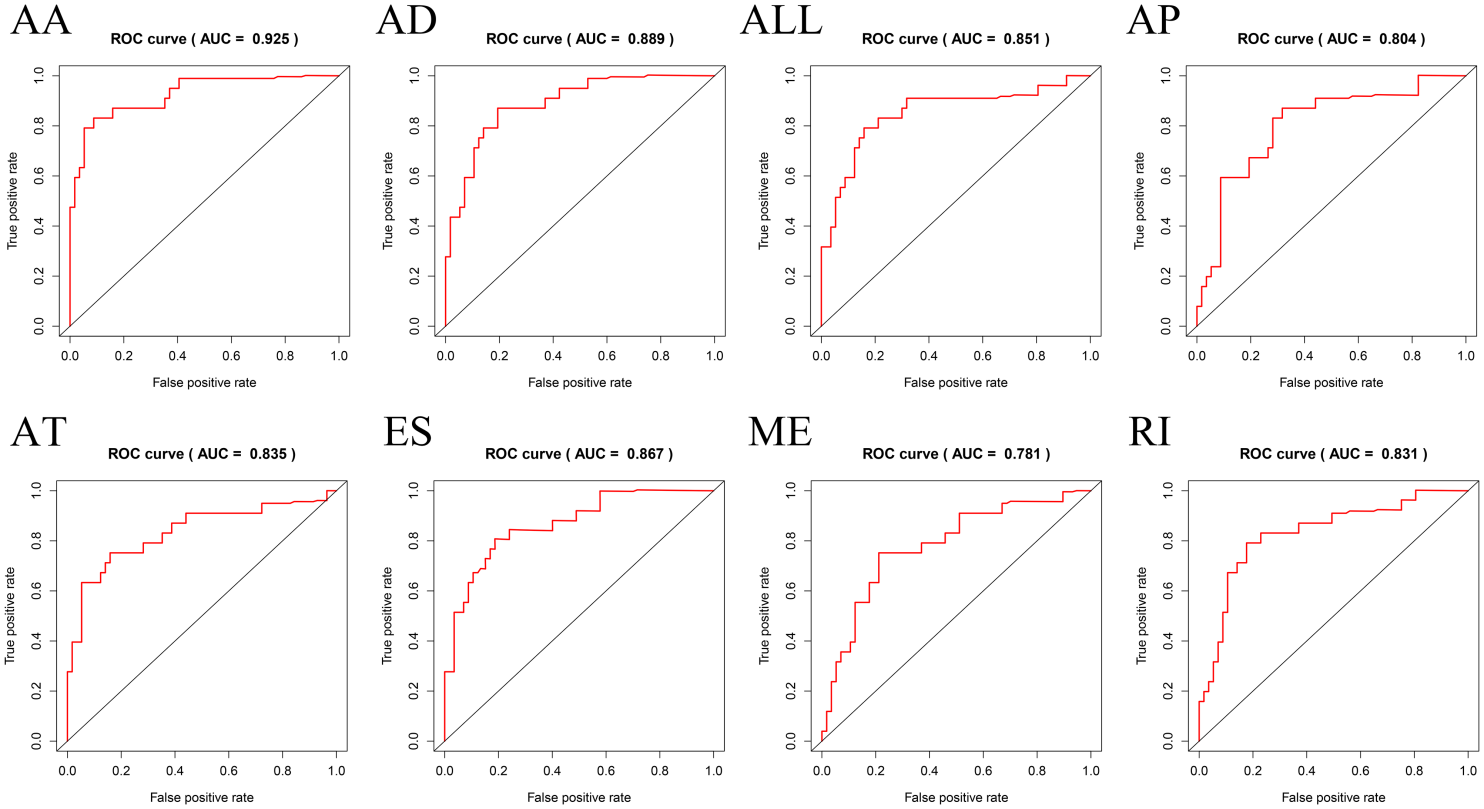
ROC curves of patients in the high‐ and low‐risk groups.

Different AS types were combined to construct prognostic models for each of the seven AS types and an overall AS prognostic model. The AS events were again filtered using multivariate Cox/LASSO regression (Figures S1 and S2), leaving a total of 41 prognosis‐associated AS events (Table 1) to prevent event overfitting. The samples were categorized into high‐ and low‐risk groups based on the mean risk values. The risk curves of different prognostic models were plotted using R. The higher the risk value, the lower was the patients' survival rate (Figures S1 and S2). The survival curves of different prognostic models were constructed using the survivor.R package^22^ to investigate the prognostic values of these models. All eight models had good prognostic values, and AA was most strongly associated with prognosis (*p* = 3.631e−11) (Figure 4). According to the results of the ROC analysis, the larger the area under the curve (AUC), the more accurate would be the prognostic model; the AA model (AUC = 0.925) had the highest prognostic value for predicting MPM (Figure 5).

**TABLE 1:** Overall survival by LASSO/multivariate Cox analysis of prognosis‐related variable shear events

AS event	AS Types	Coef	HR	HR.95 L	HR.95H	*p* value
PTPN7|9405|AA	AA	−16.4231	7.37E‐08	1.41E‐10	3.86E‐05	2.72E‐07
ARL5A|55,593|AT	AT	−32.3272	9.13E‐15	1.01E‐20	8.28E‐09	3.86E‐06
KANSL3|54,543|ES	ES	−8.55493	0.000193	2.87E‐06	0.012915293	6.69E‐05
INO80E|36,010|ES	ES	−2.72802	0.065349	0.01674	0.255110399	8.64E‐05
AGTRAP|668|ES	ES	−26.5163	3.05E‐12	4.06E‐18	2.29E‐06	0.000122
C5orf38|71,503|AT	AT	−3.73193	0.023947	0.003358	0.170773829	0.000197
LTC4S|74,924|AA	AA	−50.9154	7.72E‐23	1.60E‐34	3.72E‐11	0.000208
GEMIN6|53,288|AD	AD	−13.3838	1.54E‐06	7.13E‐10	0.003326874	0.000634
ACOXL|54,941|AT	AT	−2.74687	0.064128	0.012799	0.321300648	0.000835
ARL5A|55,593|AT	ALL	−22.9286	1.10E‐10	1.40E‐16	8.66E‐05	0.000931
IL32|33,418|AA	AA	−4.27077	0.013971	0.001066	0.183134759	0.001142
PKMYT1|33,330|AA	AA	−6.30277	0.001831	3.63E‐05	0.092452858	0.001633
MORC4|89,814|AD	AD	−7.19528	0.00075	8.20E‐06	0.068658607	0.001794
MSS51|12,150|AA	AA	−29.2668	1.95E‐13	1.10E‐21	3.44E‐05	0.00252
POLR2H|67,943|AA	AA	13.73347	921236.6	113.109	7,503,174,698	0.002798
TMEM33|69,137|RI	RI	−10.032	4.40E‐05	5.91E‐08	0.032739853	0.002946
PKMYT1|33,330|AA	ALL	−4.94584	0.007113	0.000271	0.186437805	0.002999
DUT|30,485|AP	AP	3.904597	49.63006	3.392975	725.9536336	0.004338
TBC1D7|75,381|ES	ES	−6.01096	0.002452	3.73E‐05	0.161100953	0.004878
TMC7|34,279|AT	ALL	−3.58995	0.0276	0.002184	0.348767234	0.00554
ACOXL|54,942|AT	ALL	2.780201	16.12226	2.230998	116.5071299	0.005866
MBD3|46,525|RI	RI	12.82166	370148.9	39.66551	3,454,139,960	0.005976
COPZ2|120,285|ES	ALL	3.867227	47.80962	3.027093	755.1004487	0.006021
EPN1|52,138|AA	AA	−12.3437	4.36E‐06	6.35E‐10	0.029901776	0.006169
EPS15L1|48,158|AT	AT	−5.14956	0.005802	0.000126	0.267389956	0.008417
PLSCR1|67,169|ES	ES	−7.04999	0.000867	3.85E‐06	0.195185382	0.010736
TTC13|10,258|ME	ME	2.245038	9.440775	1.630633	54.65867847	0.012221
MEIS3|50,645|AA	AA	1.965686	7.139811	1.479656	34.45185452	0.01437
RPL13|392,312|AD	AD	5.833494	341.5498	2.723692	42830.20911	0.01796
MLST8|33,226|AA	AA	−14.6293	4.43E‐07	1.65E‐12	0.118941789	0.021802
ZNF140|204,379|ME	ME	−5.46178	0.004246	3.94E‐05	0.458065023	0.022204
SEC16A|88,181|AA	AA	6.920263	1012.587	2.664695	384783.7242	0.02241
ROMO1|59,225|RI	RI	8.891291	7268.394	2.885455	18308912.13	0.02607
SDR39U1|27,012|ME	ME	−1.36389	0.255663	0.073959	0.883787348	0.031148
SRPK2|81,282|AT	AT	12.33208	226858.6	2.816276	18,274,066,785	0.032386
SMPD4|55,291|ME	ME	2.817861	16.741	1.262212	222.0397519	0.032637
GCFC2|54,161|AD	AD	−9.06928	0.000115	2.58E‐08	0.514822037	0.034449
ZFP64|59,808|AP	AP	4.577718	97.29208	1.151712	8218.849812	0.043139
TRAPPC2|88,516|RI	RI	3.184589	24.15735	1.092294	534.2676796	0.043816
TIGD6|74,053|AD	AD	1.824516	6.199792	1.024474	37.51918468	0.047001
XRCC4|72,698|AA	AA	−1.18247	0.30652	0.095302	0.985860944	0.047272

### 3.3 Independent prognosis AS models

**FIGURE 6:**
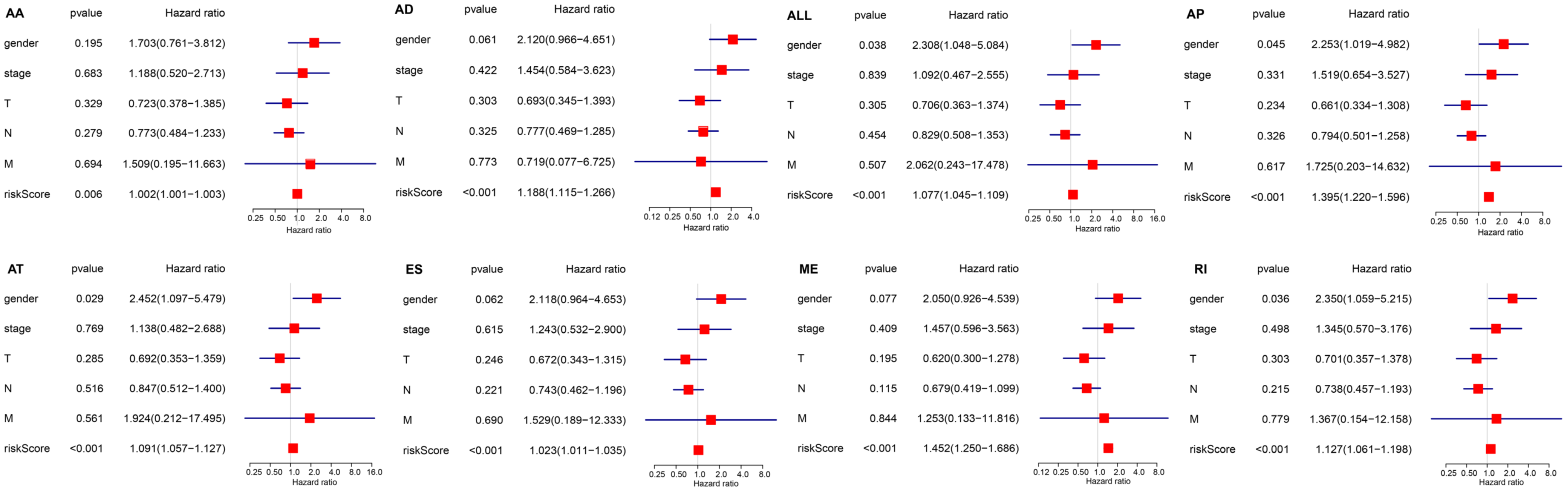
Independent prognostic analysis of AS prognostic models. The figure shows the results of the multivariate independent prognostic analysis of different AS prognostic models.

The prognostic models were analyzed for multiple clinical factors using clinical data from the TCGA database. The AS prognostic model could be regarded as an independent prognostic factor for MPM when both univariate and multivariate analyses demonstrated a *p* value of <0.05. All eight AS models were independent prognostic factors for MPM (Figure 6).

### 3.4 SF–AS interaction network

**FIGURE 7:**
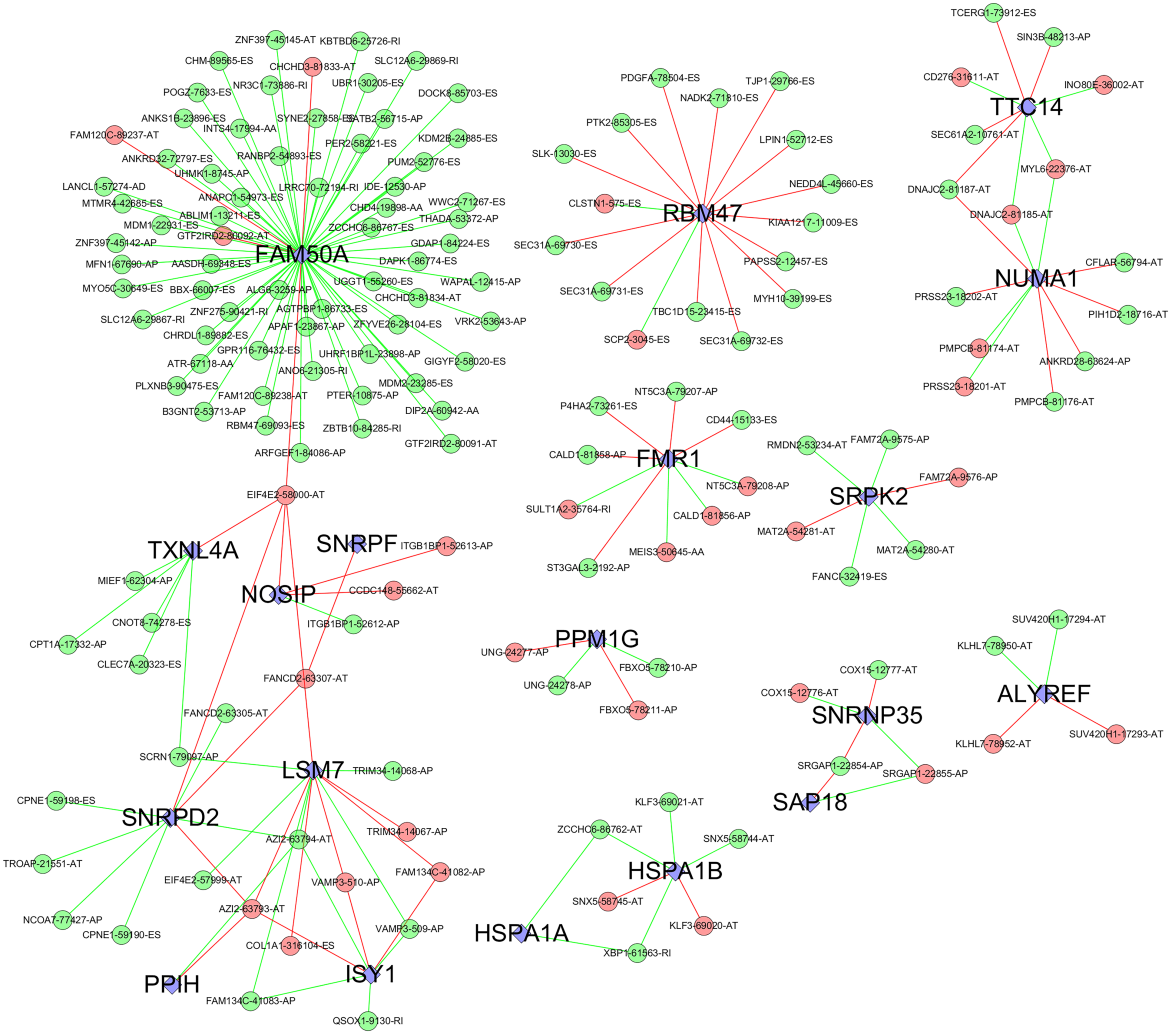
AS–SF interaction network diagram.

The SF expression values were obtained from TCGA RNA‐seq expression profile data. The R software was used to perform the Spearman test and analyze the *p*‐values of the AS of SF expression values (log2FPKM values) with the prognostic correlation obtained using the one‐way Cox analysis. When r was >0.6 and Spearman *p* value was <0.001, SF was considered to be associated with AS events. In the final results, 19 SFs (e.g., FAM50A and RBM47) were visualized using Cytoscape v.3.7.1 (Figure 7). According to the correlation and Cox analyses, the closer the SF proximity to the center of the network, the higher is the probability that it is prognostically meaningful for MPM. This helps in identifying relevant SFs and provides further directions for improving MPM prognosis.

### 3.5 Core MPM prognosis‐associated SF and SF‐enriched pathways

**FIGURE 8:**
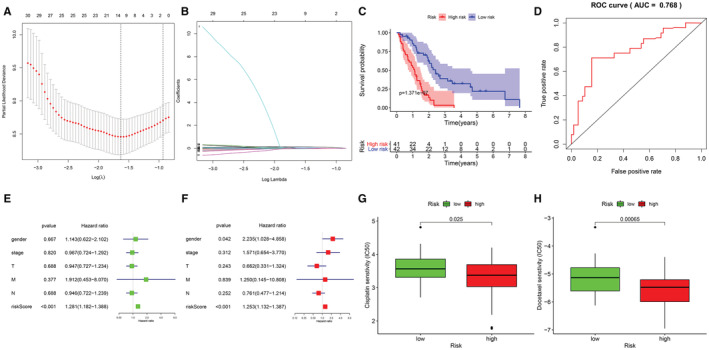
Independent prognostic models for the SFs FMP1 and PPM1G. (A and B) show the results of the LASSO/multivariate Cox analysis. (C and D) show the Kaplan–Meier and ROC curves for OS between patients in the high‐ and low‐risk groups according to the SF prognostic model. (E and F) show the results of univariate Cox and multivariate Cox analyses, where the risk scores can be an independent prognostic factor for MPM. (G and H) represent a boxplot of the difference in drug sensitivity to cisplatin and docetaxel in the high‐ and low‐risk groups, respectively.

Of the 19 SFs obtained from the SF–AS regulatory network, 11 SFs remained after screening using analysis of variance (unpaired *t*‐test) (*p* < 0.05). Multifactorial LASSO/Cox regression analyses finally selected FMP1 and PPM1G as the two SFs that were most strongly associated with MPM prognosis (Table S1). Survival analysis and ROC analysis demonstrated that the two SFs FMP1 and PPM1G had a better prognostic value among other SFs (*p* = 1.371e − 07, AUC = 0.768, Figure 8). In multivariate‐independent prognostic analysis, the SF prognostic model was an independent prognostic factor for MPM (Figure 8). Furthermore, drug sensitivity analysis of cisplatin revealed that patients with MPM and higher risk values had lower drug sensitivity (*p* = 0.025, Figure 8).

GSEA analysis can be used to investigate individual gene enrichment pathways and establish pathways and functions in which genes may be involved using large amounts of data. In our study, the gene expression profiles obtained from TCGA were used as GSEA background analysis data, which were classified into high‐ and low‐expression groups, to predict the specific pathways in which the two SFs FMP1 and PPM1G affected MPM prognosis. FMP1 and PPM1G were primarily enriched in several signaling pathways, such as TGF‐β, JAK–STAT, and RIG‐I‐like‐receptor (Table 2).

**TABLE 2:** GSEA enrichment pathway of splicing factors FMP1 and PPM1G

NAME	ES	*p*‐value	Group
KEGG_PROTEIN_EXPORT	0.753822	<0.001	FMR1‐HIGH
KEGG_VALINE_LEUCINE_AND_ISOLEUCINE_DEGRADATION	0.594927	0.001332	FMR1‐HIGH
KEGG_RNA_DEGRADATION	0.582613	<0.001	FMR1‐HIGH
KEGG_UBIQUITIN_MEDIATED_PROTEOLYSIS	0.512201	<0.001	FMR1‐HIGH
**KEGG_RIG_I_LIKE_RECEPTOR_SIGNALING_PATHWAY**	**0.509803**	**<0.001**	**FMR1‐HIGH**
**KEGG_JAK_STAT_SIGNALING_PATHWAY**	**0.434675**	**0.001101**	**FMR1‐HIGH**
KEGG_SPLICEOSOME	0.410689	0.004566	FMR1‐HIGH
KEGG_NEUROACTIVE_LIGAND_RECEPTOR_INTERACTION	−0.28096	<0.001	FMR1‐LOW
KEGG_FOCAL_ADHESION	−0.37563	<0.001	FMR1‐LOW
**KEGG_TGF_BETA_SIGNALING_PATHWAY**	**−0.38041**	**<0.001**	**FMR1‐LOW**
KEGG_VASCULAR_SMOOTH_MUSCLE_CONTRACTION	−0.38339	<0.001	FMR1‐LOW
KEGG_LEUKOCYTE_TRANSENDOTHELIAL_MIGRATION	−0.3853	<0.001	FMR1‐LOW
KEGG_CELL_ADHESION_MOLECULES_CAMS	−0.39206	<0.001	FMR1‐LOW
KEGG_LEISHMANIA_INFECTION	−0.39671	<0.001	FMR1‐LOW
KEGG_HYPERTROPHIC_CARDIOMYOPATHY_HCM	−0.40259	<0.001	FMR1‐LOW
KEGG_DILATED_CARDIOMYOPATHY	−0.41436	<0.001	FMR1‐LOW
KEGG_HEMATOPOIETIC_CELL_LINEAGE	−0.47273	<0.001	FMR1‐LOW
KEGG_ECM_RECEPTOR_INTERACTION	−0.62211	<0.001	FMR1‐LOW
KEGG_RIBOSOME	−0.6248	<0.001	FMR1‐LOW
KEGG_GLYCOSAMINOGLYCAN_BIOSYNTHESIS_CHONDROITIN_SULFATE	−0.63757	0.003344	FMR1‐LOW
KEGG_STEROID_BIOSYNTHESIS	0.654061	0.004301	PPM1G‐HIGH
KEGG_SPLICEOSOME	0.705826	<0.001	PPM1G‐HIGH
KEGG_RNA_POLYMERASE	0.692128	<0.001	PPM1G‐HIGH
KEGG_RIBOSOME	0.729201	<0.001	PPM1G‐HIGH
KEGG_PYRIMIDINE_METABOLISM	0.628346	<0.001	PPM1G‐HIGH
KEGG_PURINE_METABOLISM	0.489276	<0.001	PPM1G‐HIGH
KEGG_PROTEASOME	0.814298	<0.001	PPM1G‐HIGH
KEGG_PROGESTERONE_MEDIATED_OOCYTE_MATURATION	0.528318	<0.001	PPM1G‐HIGH
**KEGG_PENTOSE_PHOSPHATE_PATHWAY**	**0.583868**	**0.00655**	**PPM1G‐HIGH**
KEGG_OOCYTE_MEIOSIS	0.56482	<0.001	PPM1G‐HIGH
KEGG_ONE_CARBON_POOL_BY_FOLATE	0.651877	0.006565	PPM1G‐HIGH
KEGG_NUCLEOTIDE_EXCISION_REPAIR	0.565179	<0.001	PPM1G‐HIGH
KEGG_MISMATCH_REPAIR	0.71348	<0.001	PPM1G‐HIGH
KEGG_HUNTINGTONS_DISEASE	0.421105	<0.001	PPM1G‐HIGH
KEGG_HOMOLOGOUS_RECOMBINATION	0.635229	<0.001	PPM1G‐HIGH
KEGG_GLUTATHIONE_METABOLISM	0.532697	<0.001	PPM1G‐HIGH
KEGG_GLIOMA	0.470497	<0.001	PPM1G‐HIGH
KEGG_DNA_REPLICATION	0.837645	<0.001	PPM1G‐HIGH
KEGG_CELL_CYCLE	0.665223	<0.001	PPM1G‐HIGH
KEGG_BASE_EXCISION_REPAIR	0.698721	<0.001	PPM1G‐HIGH

## 4 DISCUSSION

The development of high‐throughput sequencing has led to recent advances in the study of AS. Studies on MPM and AS events are scarce, and all of them suggest that the occurrence of different AS events affect the formation of mRNA heterodimers, thereby affecting the development of MPM.^27^, ^28^, ^29^, ^30^ Langerak et al. (1992) revealed that platelet‐derived growth factor (PDGF), a chain in MPM cell lines, can undergo various AS events and that MPM cells primarily produce mRNA without exon six‐derived sequences in the PDGF A‐chain.^31^ Subsequently, Morrison et al. found that the BAP1 mutation C.2054A > T (p.Glu685Val) creates new splicing in MPM cell lines and thus affects mRNA expression.^32^ Dong et al. demonstrated for the first time that different CDK4 splice variants are expressed at different levels in the lungs of MPM‐affected patients and in those of normal individuals by analyzing MPM bulk expression microarrays.^33^ With the development of high‐throughput sequencing, Bueno et al. performed a comprehensive array analysis of 216 MPM samples in 2016 and identified 177 splicing events. They demonstrated that these AS events are differentially expressed in SF3B1 mutants and wild types.^34^ However, the association of AS events with MPM prognosis has not been elucidated.

The present study used the TCGA data, RNA‐seq profiles of 84 MPM cases from the TCGA SpliceSeq database, clinical data, and AS percentage PSI data to investigate the effect of AS events and SF on the prognosis of MPM and construct eight distinct AS prognostic models of MPM and SF–AS regulatory network using the LASSO/Cox regression analysis. Furthermore, we performed a different analysis of SF in the network using the RNA‐seq data of SF in the TCGA database to explore the molecular mechanisms of SFs in the development of MPM. The LASSO/Cox regression analysis of the high‐ and low‐risk groups helped in the identification of the SF that was most strongly associated with MPM prognosis. The analysis revealed that the SFs FMR1 and PPM1G were most strongly associated with MPM prognosis. Drug sensitivity analysis suggested that FMR1 and PPM1G are associated with resistance to multiple MPM chemotherapeutic agents, including cisplatin. To further investigate the mechanisms by which FMR1 and PPM1G acquire MPM resistance, we predicted the pathways of FMR1 and PPM1G convergence in MPM using GSEA analysis. The results showed that FMP1 and PPM1G might acquire MPM resistance through TGF‐β, JAK–STAT, RIG‐I‐like‐receptor, pentose‐phosphate, and other signaling pathways. This enables the advancement of drug resistance in patients with MPM.

The fragile X mental retardation protein (FMR1) is an RNA‐binding protein that is generally believed to regulate the expression of genes. This protein is essential for neuronal growth and synaptic plasticity through transcription control.^35^, ^36^ Existing studies indicate that its G‐quartet motif can act as a control element (i.e., SFs) of AS, regulating the occurrence of AS events.^37^, ^38^ This is consistent with the findings of our Spearman test analysis. However, no study has yet described the role of FMR1 in MPM. Based on the results of our analysis, we can speculate that FMR1 affects patients with MPM prognosis through several signaling pathways, such as TGF‐β, JAK–STAT, and RIG‐I‐like‐receptor, or by regulating the occurrence of AS events. Protein phosphatase magnesium‐dependent 1 (PPM1G), a Ser/Thr protein phosphatase in the PP2C family, can affect AS by regulating the dephosphorylation of pre‐mRNA SFs.^39^, ^40^ When PPM1G is bound to molecules, including 7SK RNA, it prevents the assembly of SFs7SK snRNP and maintains transcriptional elongation.^41^ This is another indication that PPM1G can affect the presence of AS events. Additionally, PPM1G may affect the prognosis of MPM primarily by affecting the pentose‐phosphate pathway or by affecting the AS events. However, these are speculations based on bioinformatics analysis, and experimental evidence is required.

## 5 CONCLUSION

Our study analyzed AS and prognostic values of SF for MPM as well as the potential pathways by which SF affects MPM. However, there are several limitations in our study: (1) the analytical results were obtained through extensive data analysis, which required the experimental evidence for specificity. (2) as MPM is a rare malignancy, the existing sample size of the MPM cohort was small, indicating bias in the results. (3) the novel therapeutic agent pemetrexed for MPM is yet to be analyzed for drug sensitivity.

## AUTHORS' CONTRIBUTIONS

YJ analyzed the data and wrote the manuscript; CDZ collected and analyzed the data; YC and SYZ analyzed the data; YPH laid out of figures; JH designed the study and revised the manuscript. All authors read and approved the final manuscript.

## FUNDING INFORMATION

This study was funded by the Health Commission of Mianyang City (grant number 202001).

## CONFLICT OF INTEREST

The author reports no conflicts of interest in this work.

## ETHICS APPROVAL AND CONSENT TO PARTICIPATE

Not applicable.

## CONSENT FOR PUBLICATION

Not applicable.

## Supporting information


Figure S1

Figure S2

Table S1

## Data Availability

The datasets used and/or analyzed during the current study are available from the corresponding author on reasonable request.
