# Supplementary figures and images for: Prognostic risk assessment model for alternative splicing events and splicing factors in malignant pleural mesothelioma
Source: Cancer Med. 2022 Aug 28;12(4):4895–906. doi: 10.1002/cam4.5174 (PMC9972025; doi:10.1002/cam4.5174)

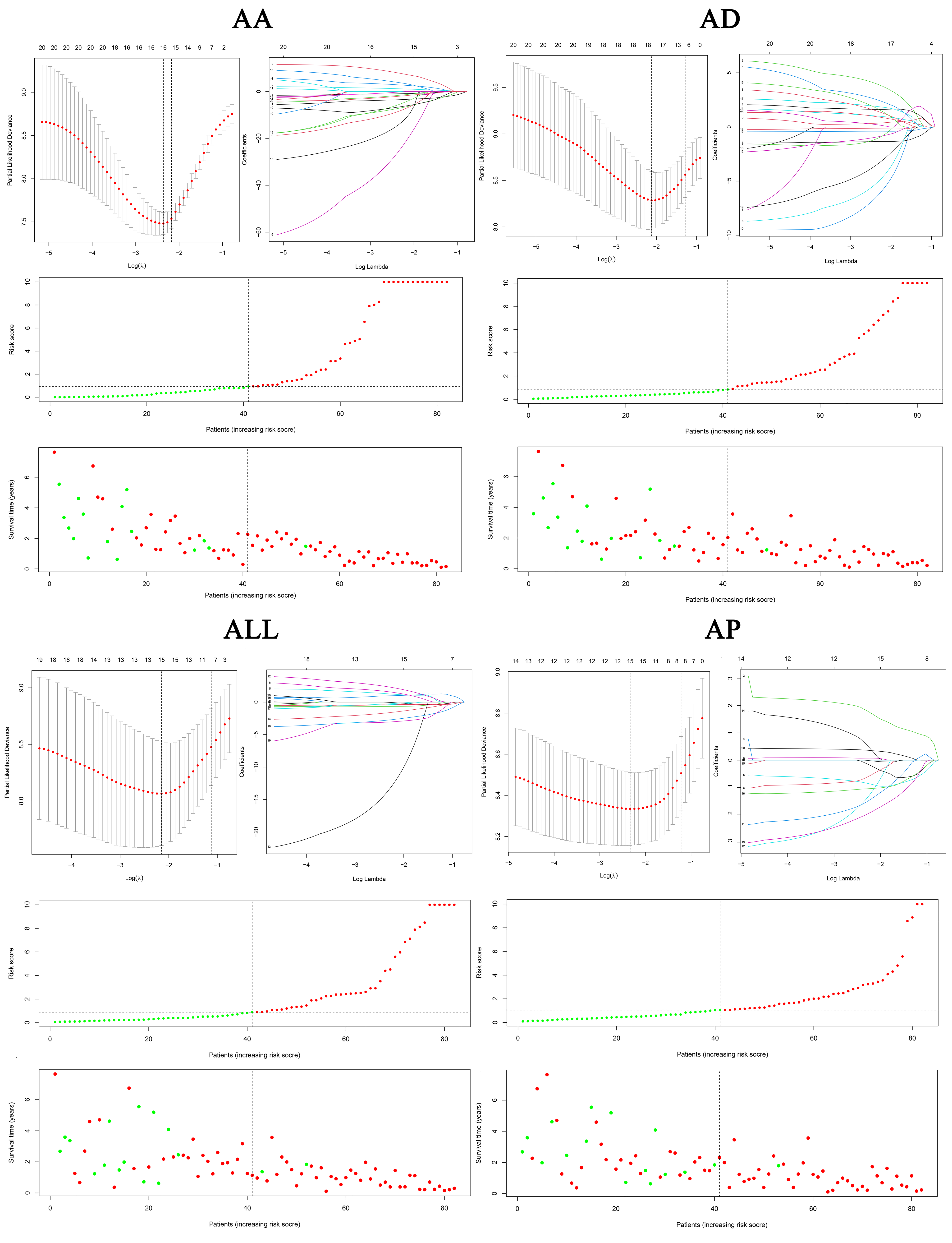

Supplement: Supplementary file 1 — Figure S1 [file CAM4-12-4895-s001.tif]

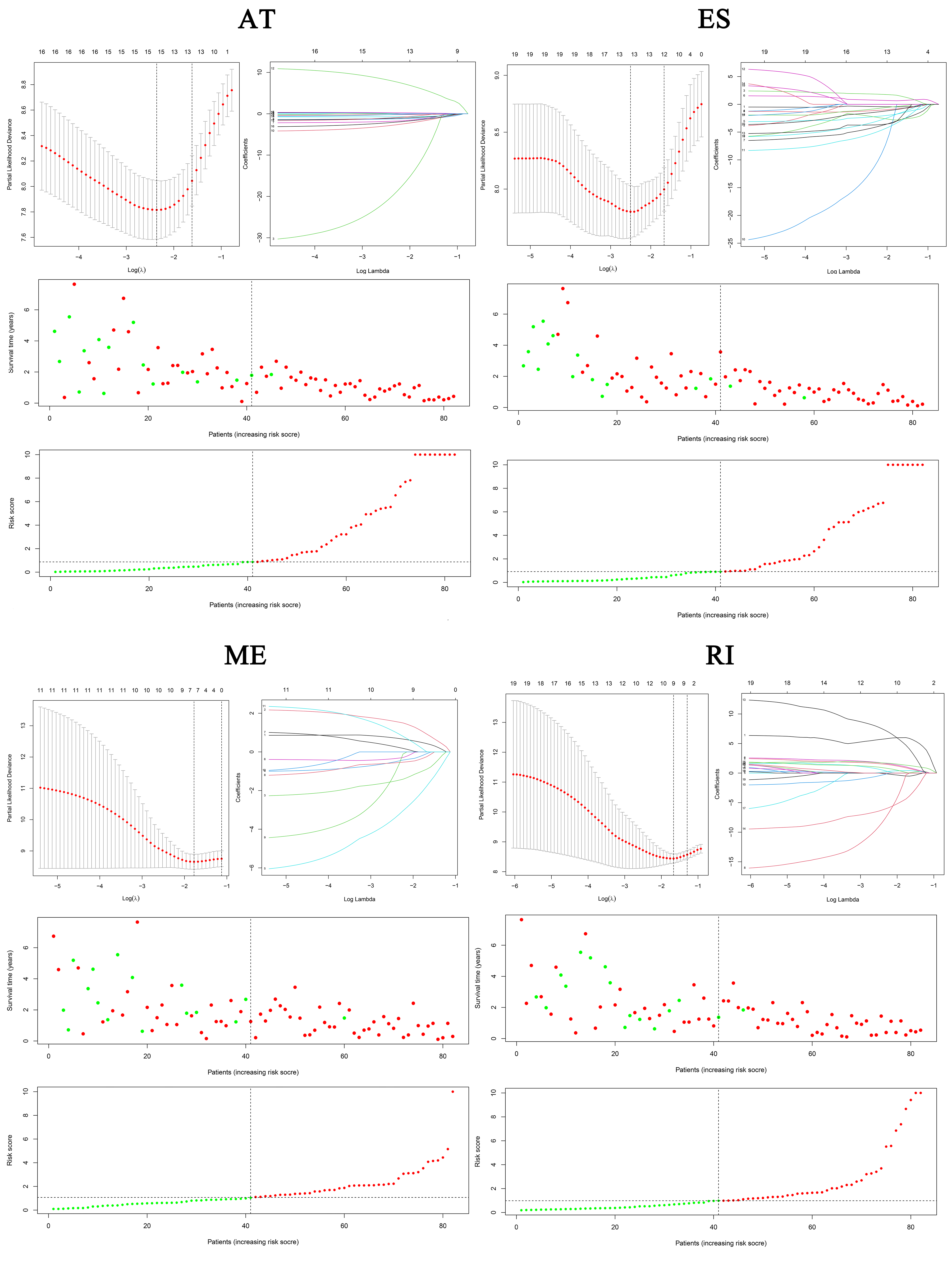

Supplement: Supplementary file 2 — Figure S2 [file CAM4-12-4895-s002.tif]
